# Transatrial left-ventricular cannulation for a shaggy aorta in aortic arch surgery: a case report

**DOI:** 10.1186/s44215-026-00260-7

**Published:** 2026-05-12

**Authors:** Atsutaka Aratame, Masanori Sakaguchi, Ryosuke Ieguchi, Toshio Baba

**Affiliations:** Department of Cardiovascular Surgery, Bell-Land General Hospital, 500-3, Higashiyama, Naka-ku, Sakai city, Osaka 599-8247 Japan

**Keywords:** Transatrial left-ventricular cannulation, Shaggy Aorta, Thromboembolism

## Abstract

**Background:**

Ascending aortic cannulation is the standard arterial inflow strategy for cardiovascular surgery that requires cardiopulmonary bypass. However, this approach can be problematic in patients with extensive atheromatous disease of the ascending aorta, commonly referred to as shaggy aorta, because of the increased risk of thromboembolic complications. Therefore, alternative cannulation strategies are required in such high-risk settings.

**Case presentation:**

A 75-year-old woman presented with a 55-mm distal aortic arch aneurysm accompanied by an extensively shaggy aorta involving the ascending aorta. Ascending aortic arch replacement using the frozen elephant trunk technique was performed, and the transatrial left ventricular cannulation technique for arterial inflow. Cardiopulmonary bypass was established while preserving the spontaneous cardiac activity with epicardial pacing during cooling. Additional embolic prevention strategies include temporary retrograde cerebral perfusion before selective antegrade cerebral perfusion and controlled blood evacuation via the femoral artery during reperfusion. The operative, cardiopulmonary bypass, cardiac arrest, and circulatory arrest times were 326, 185, 91, and 61 min, respectively. The postoperative course was uneventful, and no thromboembolic complications, including cerebral infarction, were observed. Postoperative imaging confirmed the complete exclusion of the aneurysm without endoleaks.

**Conclusion:**

Thoracic aortic aneurysm surgery in patients with a shaggy ascending aorta carries a substantial risk of embolism. The present case demonstrates that transatrial left ventricular cannulation combined with multiple embolic prevention strategies might be a safe and effective option for aortic arch surgery in this high-risk population.

## Background

Ascending aortic cannulation is the standard method of arterial inflow in cardiovascular surgery requiring cardiopulmonary bypass (CPB). However, this approach can be challenging in patients with aortic dissection, extensive calcification, or atheromatous diseases of the ascending aorta.

Schoeneich et al. reported a transatrial left ventricular (TA-LV) cannulation technique in patients with aortic dissection, in which an arterial cannula was inserted into the left ventricle via the right superior pulmonary vein and left atrium to establish arterial perfusion [1].

In the present report, a case of aortic arch aneurysm with a shaggy aorta involving the ascending aorta that was successfully treated using TA-LV cannulation is described.

## Case presentation

**Fig. 1:**
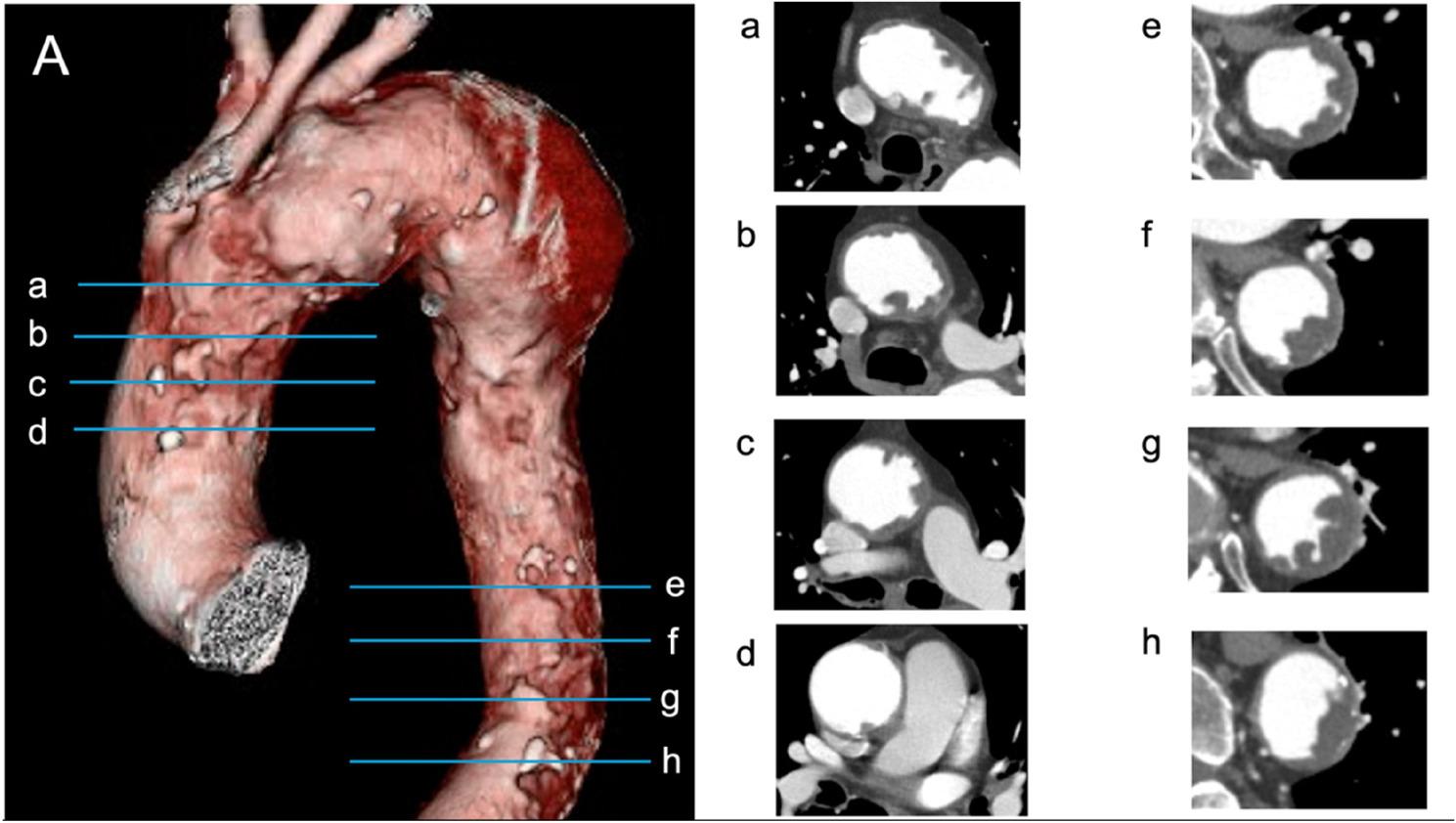
Contrast-enhanced computed tomography revealed an aortic arch aneurysm with extensive irregular intraluminal material, consistent with a shaggy aorta, extending from the ascending to the descending aorta

A 75-year-old woman presented with a 55-mm distal aortic arch aneurysm and an extensive shaggy aorta. Her medical history included hypertension and mild cerebral infarction, which had hardly affected her physical abilities. Shaggy aortic changes extend from the ascending to the descending aorta (Fig. 1). A staged TEVAR strategy carries a substantial risk of aeroembolism due to stent-graft device delivery and guidewire manipulation. Furthermore, simple aneurysmal exclusion poses the risk of recurrent laryngeal nerve injury and oesophageal damage. Regarding balance, the frozen elephant trunk technique was considered the most appropriate and comprehensive treatment strategy for this patient.

### Surgical procedure

**Fig. 2:**
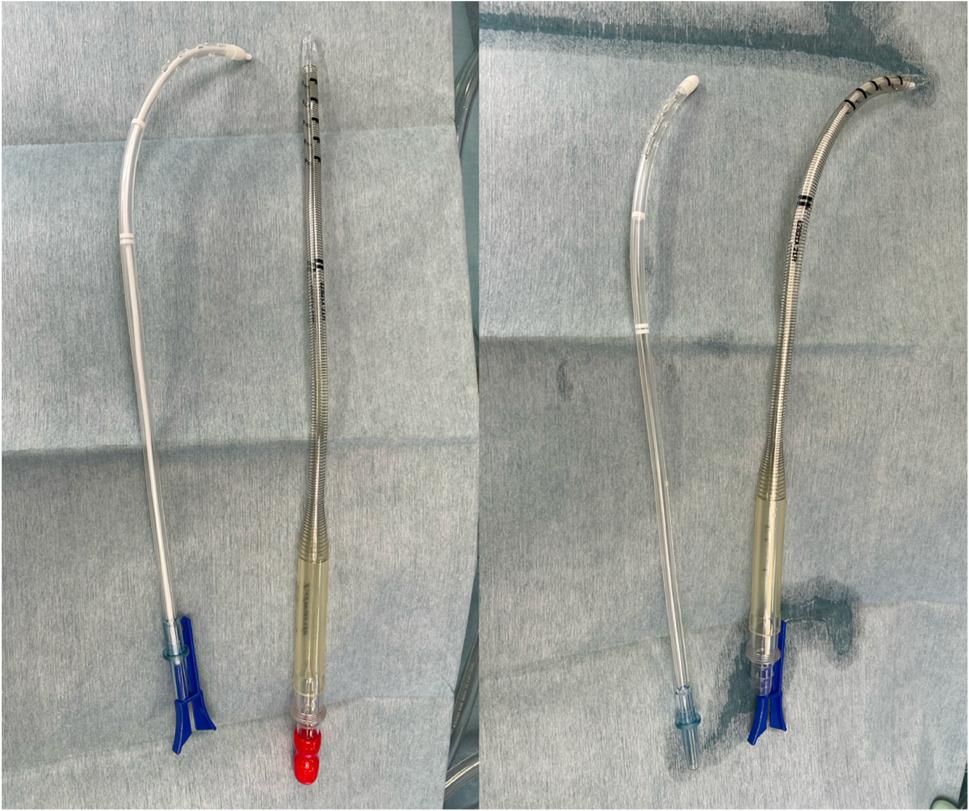
The curved plastic introducer attached to the Williams vent catheter facilitates the safe and easy insertion of the arterial cannula

**Fig. 3:**
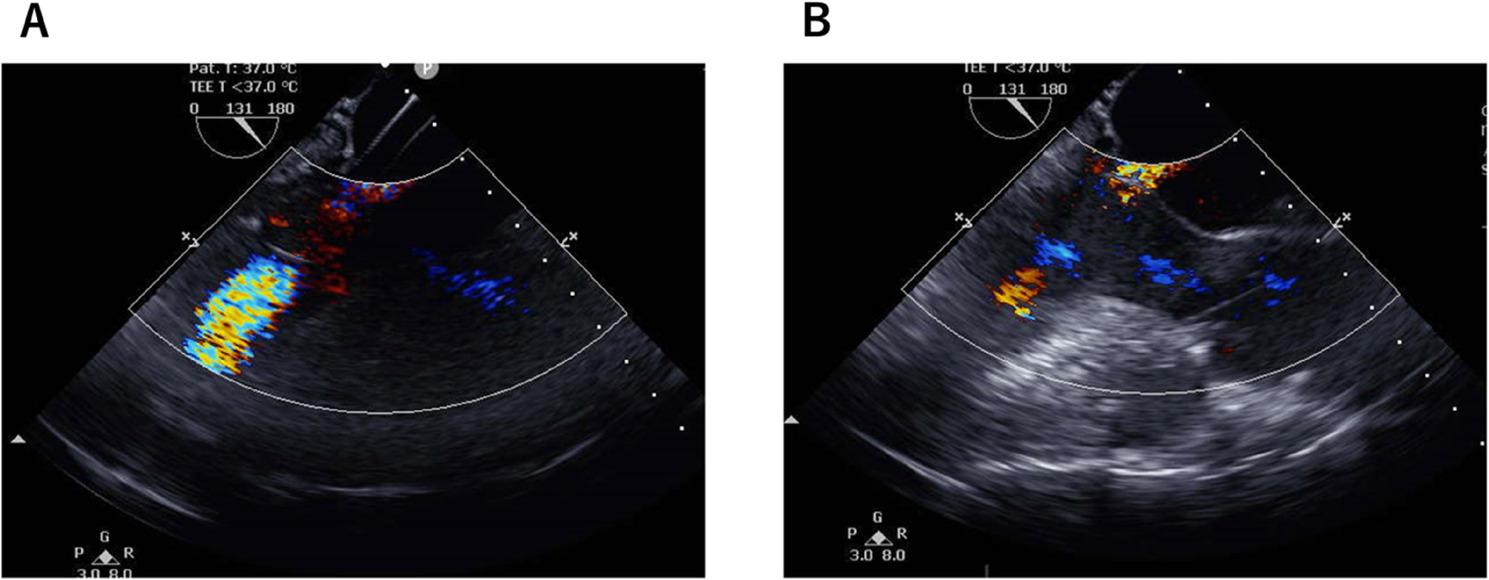
**A** Transoesophageal ultrasonography colour doppler imaging demonstrated a mosaic flow pattern at the tip of the arterial cannula, indicating a turbulent high-velocity perfusion jet. **B** It was confirmed that cannula interference did not result in severe mitral regurgitation or left ventricular outflow tract obstruction

**Fig. 4:**
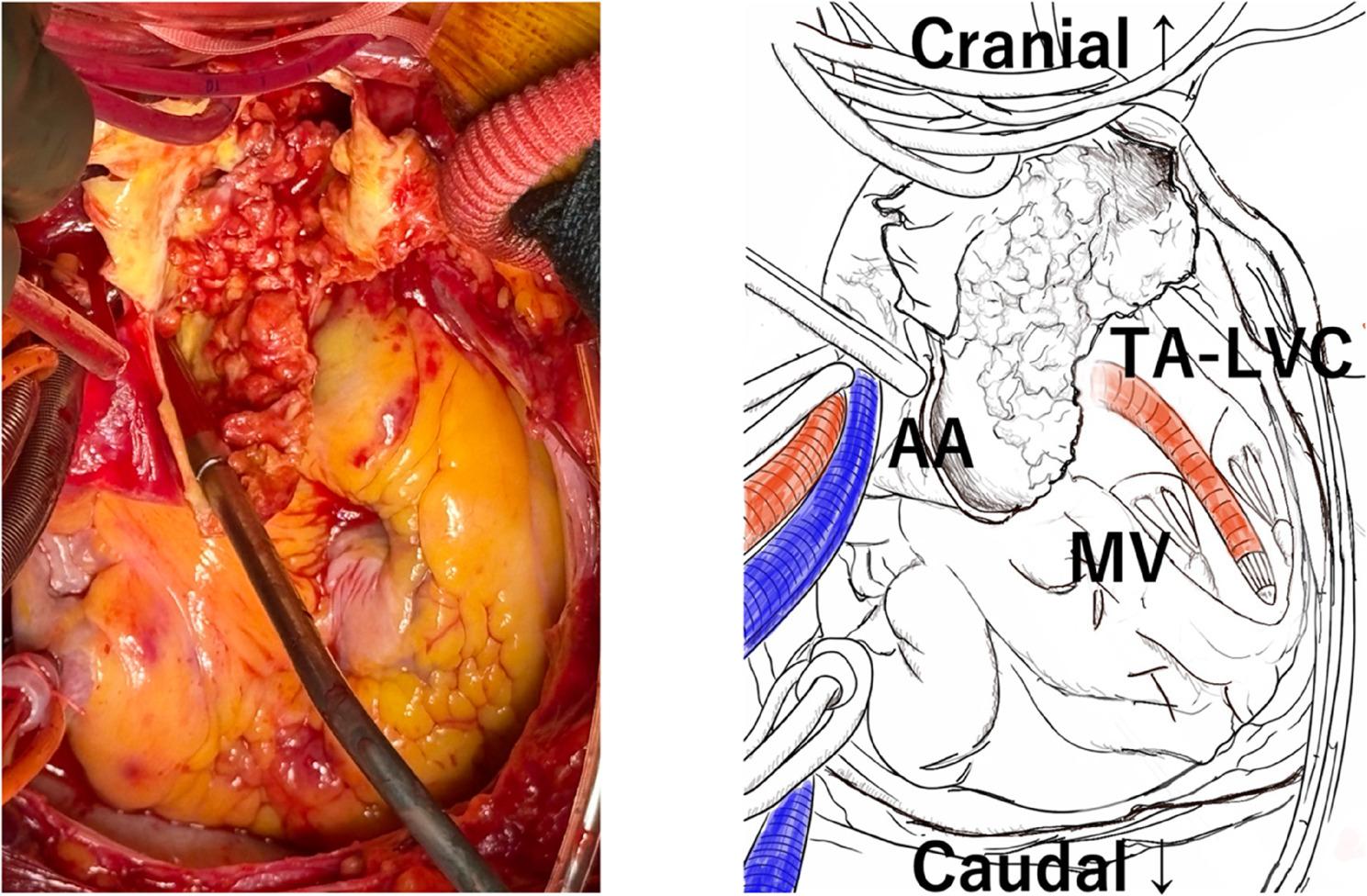
Intraoperative inspection demonstrated extensive and friable atheromatous plaques extending from the ascending aorta to the aortic arch. The right figure presents a schematic representation of the intraoperative findings, illustrating the planned position of transatrial left-ventricular cannulation. AA ascending aorta, TA-LVC tranastrial left-ventricular cannula, MV mitral valve

A Swan-Ganz catheter was inserted preoperatively to record the pulmonary artery pressure during surgery. Following median sternotomy and systemic heparinisation, CPB was performed via the left atrium using a 21Fr arterial soft cannula (EZ Glide; Edwards Lifesciences) described in the TA-LV cannulation technique. Although insertion of the perfusion cannula follows a technique similar to that used for left ventricular vent placement via the right upper pulmonary vein and left atrium, careful manipulation is essential. A curved plastic introducer attached to a Williams vent catheter (Mera, Japan) was routinely used as it allows safe and reliable cannula placement (Fig. 2). The superior and inferior vena cavae were cannulated using a common venous cannula. A pump flow of 2.4 L/min/m² was maintained throughout the procedure. The LA-LV cannula position was carefully monitored using the Swan-Ganz catheter data and transesophageal echocardiography (Fig. 3). The relationships between the cooling rate, systemic blood pressure, and pulmonary artery pressure are shown in Table 1. As body temperature decreases, the risk of ventricular fibrillation increases. Therefore, once the pharyngeal temperature reached 32 ℃, the rate of cooling was reduced, and epicardial back-up pacing VVI 50 ppm was initiated. Additionally, during systemic cooling, the mean systemic arterial pressure was maintained between 40 and 50 mmHg, while CPB flow was preserved to prevent pulmonary oedema and left ventricular overdistension in the event of ventricular fibrillation. Circulatory arrest was induced once moderate hypothermia (pharyngeal temperature, 27 °C) was achieved. Myocardial protection was achieved using retrograde cardioplegia, and selective cerebral perfusion was initiated. The intraoperative findings revealed extensive friable atheromatous plaques extending from the ascending aorta to the aortic arch (Fig. 4). A frozen elephant trunk prosthesis (35 mm×12 cm, Frozenix^®^, Japan Lifeline, Tokyo, Japan) was deployed from the level of the left common carotid artery bifurcation. A four-branched J Graft (J Graft^®^, Japan Lifeline, Tokyo, Japan) was anastomosed to the distal aorta. After completion of the distal anastomosis, controlled blood evacuation via the exposed left femoral artery was intentionally performed to prevent distal embolisation, followed by resumption of arterial inflow through the side branch of the graft. The previously placed TA-LV cannula was repurposed as a left ventricular vent by connecting it to a venting suction line.

**Table 1:** Changes in Systemic and Pulmonary Artery Pressures During Cooling

Time(minutes)	Core temperature	SAP (sys/dia/mean)	PAP(sys/dia/mean)
0	36℃	81/40/57	26/11/17
10	32℃	67/33/47	32/18/25
20	30℃	49/32/40	32/18/25
30	28℃	42/30/38	21/17/20

*Abbreviations*: *SAP* systemic arterial pressure, *PA* pulmonary arterial pressure, *sys* systolic, *dia* diastolic, *mean* mean pressure

**Fig. 5:**
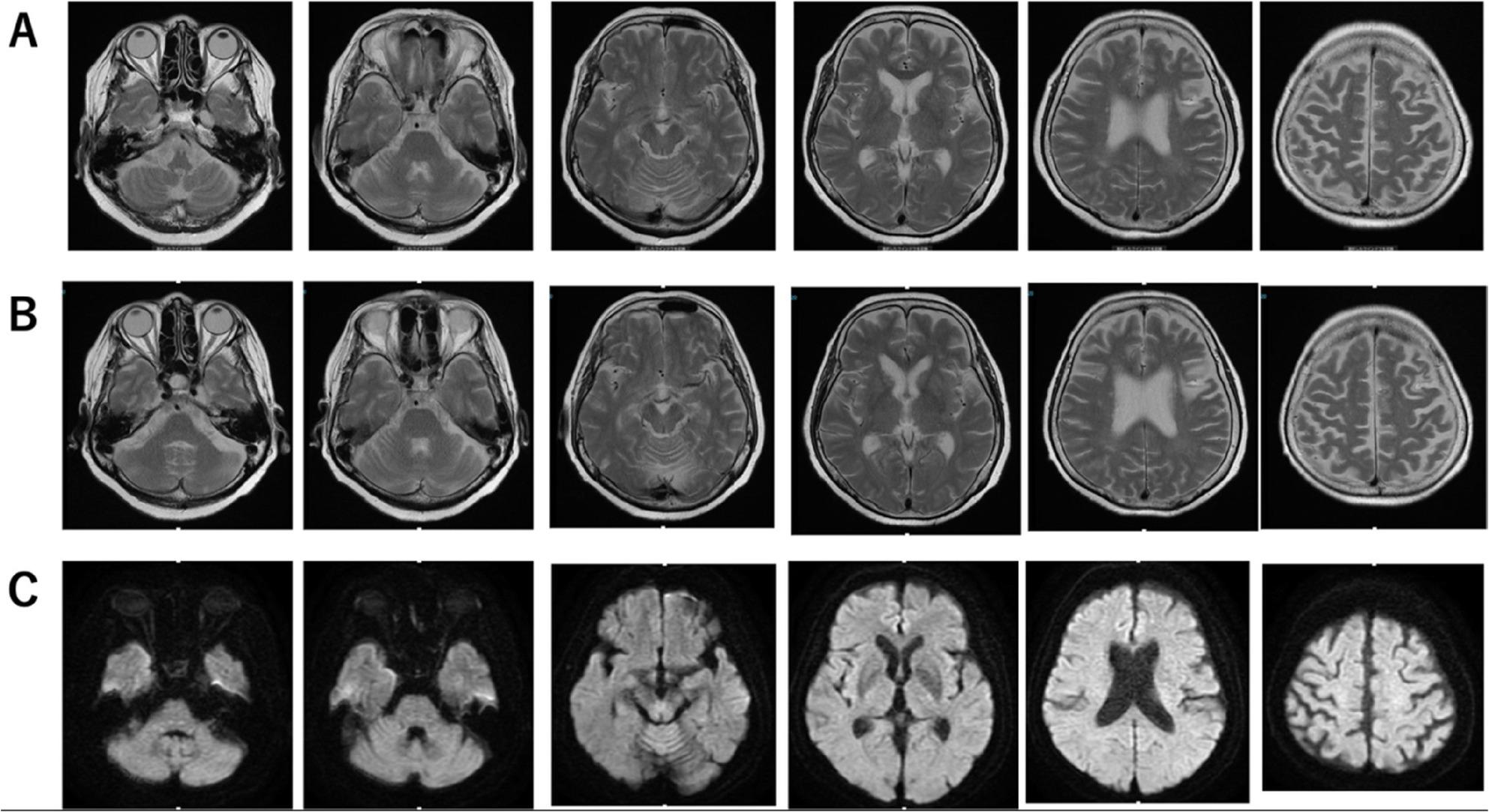
**A** Preoperative cerebral magnetic resonance imaging (MRI), T2-weighted sequence. **B** Postoperative MRI, T2-weighted sequence. **C** Postoperative MRI, diffusion-weighted imaging. Postoperative imaging demonstrated no new cerebral infarction, except for a pre-existing chronic subcortical infarct in the left frontal lobe

**Fig. 6:**
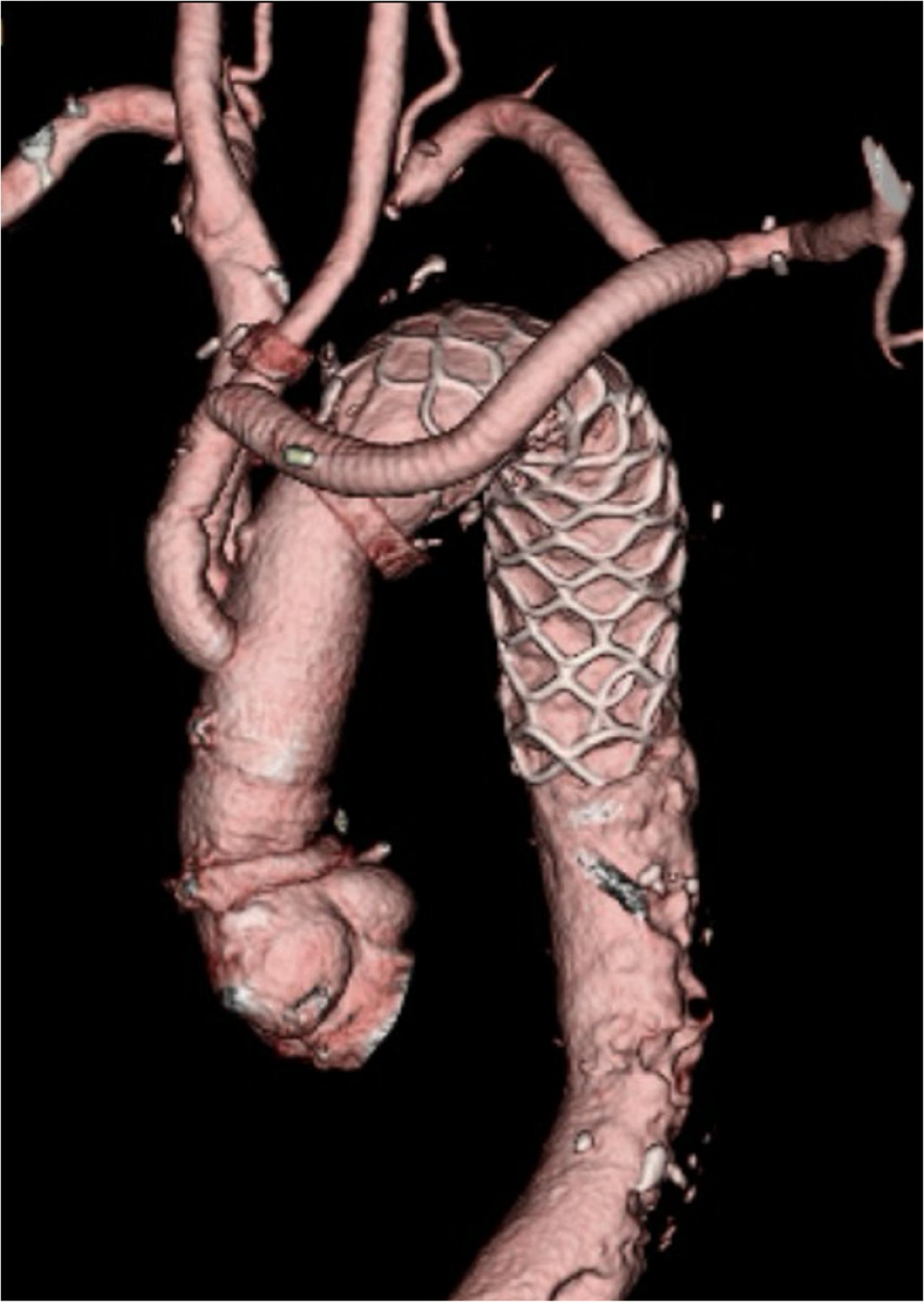
Postoperative contrast-enhanced computed tomography demonstrated complete exclusion of the aortic arch aneurysm without evidence of endoleak

The proximal anastomosis was completed, and spontaneous cardiac activity resumed. The left subclavian, common carotid, and brachiocephalic arteries were sequentially reconstructed. The operative, CPB, cardiac arrest, and circulatory arrest times were 326, 185, 91, and 61 min, respectively. The postoperative course was uneventful, with no major complications. No physiological or laboratory findings suggested a thromboembolism. The peak postoperative Creatine kinase-MB level was 9.6 ng/mL, which was within the normal range for the postoperative course. Furthermore, postoperative echocardiography demonstrated a preserved left ventricular ejection fraction and normal valvular function. Compared with the preoperative cerebral magnetic resonance imaging, postoperative cerebral magnetic resonance imaging revealed no new cerebral infarction, except for a pre-existing chronic subcortical infarct in the left frontal lobe (Fig. 5). Postoperative contrast-enhanced computed tomography confirmed complete exclusion of the aneurysm without an endoleak (Fig. 6).

## Discussion and conclusions

“Shaggy aorta” generally refers to severe, diffuse atherosclerotic involvement of the aorta, in which an irregular luminal surface with ulceration and friable atheromatous debris is associated with an increased risk of embolisation, while a standardised definition has not been established [2].

In thoracic aortic aneurysm surgery with a shaggy aorta, embolism is also among the most severe complications, with cerebral infarction having a substantial impact on prognosis. Preventive strategies involving standard ascending aortic cannulation have been proposed [3, 4]. However, in the setting of an ascending shaggy aorta, careful selection of the arterial inflow route, except for ascending cannulation, may be essential when performing thoracic aortic aneurysm surgery.

Common alternative arterial inflow routes include axillary and femoral cannulations. Surgical outcomes associated with different arterial inflow routes have been widely studied, particularly in the context of acute aortic dissection. Although several reports suggest that axillary cannulation reduces the risk of stroke compared to femoral cannulation, others have found no significant differences in stroke or early mortality, leaving the issue unresolved [5–7]. These inconsistent results may reflect the disease-specific characteristics of aortic dissection, such as false lumen thrombosis and dissection-related malperfusion.

In contrast, in patients with shaggy aortas, the focus of the discussion seemed to be somewhat different. In this setting, the presence of extensive, fragile, and irregular atheromatous plaques constitutes the primary risk factor, and a key aspect of the surgical strategy is the prevention of plaque disruption and embolisation caused by arterial cannulation and the jet flow generated during CPB. Therefore, the inflow direction and haemodynamic factors should be considered to prevent embolism.

With conventional arterial inflow from the ascending aorta, the direction of the inflow jet varies depending on the orientation of the cannula tip and the type of arterial cannula used [8]. Femoral arterial cannulation inevitably leads to retrograde perfusion of the thoracic aorta, whereas axillary artery cannulation results in retrograde flow toward the ascending aorta, particularly during diastole [9]. These haemodynamic features indicate that in cases of a shaggy ascending aorta, femoral, axillary, and ascending aortic cannulation strategies may increase the risk of embolic events through jet flow and turbulence. Alternatively, transapical perfusion may be considered as a method for maintaining physiological haemodynamics. Generally, the tip of the cannula should be positioned above the aortic valve [10], as the perfusion jet may otherwise directly impact atherosclerotic plaques. If the tip remains within the left ventricle and becomes dislodged, there is a potential risk of systemic embolisation. Additionally, myocardial injuries associated with apical puncture or cannula dislodgement should be carefully considered.

Thus, three strategies were employed to minimise the risk of embolisation.

First, careful consideration was given to the arterial perfusion strategy. To prevent embolisation from the ascending aortic atheroma most effectively, the primary goal was to preserve the physiological cardiac output as much as possible. TA-LV cannulation utilises the left ventricle as the source of systemic perfusion, thereby possibly facilitating a more physiological antegrade flow in the ascending aorta and arch vessels compared with axillary or carotid cannulation. In this context, maintaining a natural flow pattern and avoiding non-physiological perfusion of the arch vessels were considered important, which led us to select TA-LV cannulation in this case.　Epicardial pacing was used to prevent ventricular fibrillation, allowing spontaneous cardiac activity to be maintained until the target temperature was reached.

Favourable outcomes of TA-LV cannulation have been reported in patients with aortic dissection [11, 12]. Severe mitral annular calcification is a contraindication to this approach [11]. However, given the previous report of severe pulmonary oedema associated with this cannulation technique [13], conditions such as severe mitral regurgitation, severe aortic stenosis, and left ventricular outflow tract obstruction may represent relative contraindications, as these pathologies could increase the left ventricular load and consequently exacerbate pulmonary venous congestion. To mitigate these risks, the pulmonary artery pressure was continuously monitored using a Swan–Ganz catheter, and the correct positioning of the arterial cannula within the left ventricle was confirmed using transesophageal echocardiography. Additionally, based on our experience, particularly with TA-LV cannulation, preservation of spontaneous cardiac activity through cooling to a target temperature depends on two key factors: adequate venous drainage (additional drainage via the pulmonary artery or use of vacuum-assisted venous drainage) during CPB to achieve sufficient cardiac decompression and the use of epicardial pacing. Preservation of spontaneous cardiac activity is considered as important as possible, not only for maintaining physiological cardiac output but also for preventing left ventricular overdistension and increases in pulmonary artery pressure. However, if ventricular fibrillation occurs despite these preventive measures, the rate of cooling should be accelerated to achieve the target temperature as promptly as possible, and the mean systemic arterial pressure should be reduced (at around 50mmHg) as long as adequate CPB flow is preserved. Additionally, if pulmonary oedema or cardiac overdistension is anticipated, such as when pulmonary arterial pressure exceeds systemic arterial pressure, conversion to an alternative perfusion route should be considered, and the TA–LV cannula may be repurposed as a venting cannula.

Secondly, temporal retrograde cerebral perfusion was performed until selective antegrade cerebral perfusion was achieved. Even if the embolisation caused by the arterial jet from the cannula can be avoided, atheromatous debris may still enter the cervical branch vessels during aortotomy and manipulation before the establishment of selective cerebral perfusion. Its use was limited for a short period until selective cerebral perfusion was established to prevent cerebral embolic events.

Finally, during reperfusion after the completion of the distal anastomosis, controlled blood evacuation via the left femoral artery was performed to prevent embolisation of the lower extremities and abdominal organs by atheromatous debris, which was presumed to have been generated during peripheral anastomotic manipulation under circulatory arrest. A low-pressure outlet was intentionally created by opening the femoral artery to allow atheromatous debris presumed to be floating in the bloodstream to be expelled extracorporeally. The drained blood was collected via suction; therefore, no blood loss occurred.

## Conclusions

Favourable outcomes were achieved using multiple embolic prevention strategies for aortic arch aneurysms associated with a shaggy ascending aorta. The TA–LV cannulation technique is described in detail, focusing on its safety profile, potential complications, and preventive measures. TA-LV cannulation might be one of the useful perfusion methods in patients with a shaggy ascending aorta, as demonstrated in the present case.

## Data Availability

The datasets used during the current study are available from the corresponding author on reasonable request.

## Abbreviations

CPB: cardiopulmonary bypass
TA-LV: transatrial left-ventricular
